# Academic Discipline, Self-Medication, and Medication-Related Behaviors in Premenstrual Symptom Management Among Female University Students: A Cross-Sectional Study

**DOI:** 10.3390/healthcare14203465

**Published:** 2026-10-15

**Authors:** Olcay Kıroğlu, Havanur Güllü, Rozelin Aydın, İlker Ünal

**Affiliations:** 1 Department of Medical Pharmacology, Faculty of Medicine, Çukurova University, Sarıçam, 01250 Adana, Türkiye; havanur916@gmail.com; 2 Department of Bioengineering, Faculty of Engineering, Adana Alparslan Türkeş Science and Technology University, Sarıçam, 01250 Adana, Türkiye; raydin@atu.edu.tr; 3 Department of Biostatistics, Faculty of Medicine, Çukurova University, Sarıçam, 01250 Adana, Türkiye; ilkerun@cu.edu.tr

**Keywords:** premenstrual symptoms, self-medication, medication-related behavior, medication safety, complementary medicine, university students, women’s health

## Abstract

Background: Premenstrual symptoms are common among young women and may lead to repeated self-management and medication use. However, medication-related behaviors may vary across academic disciplines, and it remains unclear whether favorable medication-safety attitudes are reflected consistently in everyday behavior. Methods: This cross-sectional study evaluated medication-related, self-care, complementary, and healthcare-seeking behaviors among female undergraduate students reporting premenstrual symptoms at Çukurova University, Türkiye, between January and June 2024. Data from 479 students across five faculties were collected using a 53-item, study-specific questionnaire covering sociodemographic characteristics, symptom-management practices, healthcare-seeking behavior, medication use, self-medication, complementary and herbal product use, and medication-related attitudes. Associations between categorical variables were examined using Pearson’s chi-square test, with Cramer’s V reported as a measure of effect size. Results: Among participants, 88.1% reported regular menstrual cycles, and 30.5% reported previous education on premenstrual symptom management. Family members or friends were the most frequently reported source of advice (47.4%), followed by physicians (27.6%). Analgesics were commonly used (71.0%), 39.9% reported using medications already available at home, and 78.4% of medical students agreed that they used analgesics without physician recommendation. Herbal or aromatherapy-based approaches were reported by 29.9% of participants. Faculty-level differences were observed in medication-related behaviors, attitudes toward complementary approaches, and information-seeking patterns. Conclusions: Medical students reported more cautious attitudes toward herbal safety and informal information sources, although self-medication-related behaviors remained common. These findings support university-based health promotion strategies that emphasize practical medication-safety skills, appropriate healthcare seeking, evidence-based self-care, responsible use of complementary products, and critical evaluation of digital health information.

## 1. Introduction

Premenstrual symptoms are recurrent physical, emotional, and behavioral experiences that occur during the late luteal phase of the menstrual cycle and may affect daily functioning and quality of life. Premenstrual syndrome (PMS) refers to a clinically significant pattern of such symptoms, whereas premenstrual dysphoric disorder (PMDD) is characterized by more severe affective symptoms and functional impairment [1,2,3,4,5]. Among university students, premenstrual symptoms may coincide with academic stress, sleep disturbances, changes in dietary habits, and other lifestyle factors, potentially affecting concentration, attendance, academic performance, and social functioning [6,7,8,9,10,11].

Management of premenstrual symptoms involves a broad range of pharmacological and non-pharmacological approaches. Clinical recommendations emphasize individualized management that may include lifestyle modification, physical activity, stress reduction, psychological interventions, hormonal treatments, non-steroidal anti-inflammatory drugs, and, when clinically indicated, selective serotonin reuptake inhibitors [12,13]. At the same time, young women may independently use analgesics, previously obtained medications, herbal products, dietary supplements, or other complementary approaches. Such practices may be influenced by previous experiences, perceived symptom severity, medication accessibility, family and peer recommendations, and information obtained through digital and social media sources [14,15,16].

Self-medication is relevant from a medication-safety perspective because using medicines without professional assessment may increase the risk of inappropriate drug selection or dosing, contraindications, drug interactions, and delayed professional healthcare seeking [17,18,19,20,21,22,23]. These concerns are also relevant to complementary and herbal products. The perception that products of natural origin are inherently safe may contribute to inappropriate use, while differences in product composition, dosage, quality, and potential interactions with conventional medicines may create additional safety concerns [24,25].

Medication safety extends beyond pharmacological knowledge alone. It also involves recognizing when professional consultation is needed, considering potential benefits and risks, and making informed decisions about pharmacological and non-pharmacological options. Previous studies among university students suggest that students in health-related disciplines may demonstrate greater awareness of medication safety; nevertheless, self-medication and medicine use without professional guidance remain common even among students receiving health-related education [17,18,20,21,22,26,27]. Because the present questionnaire was not a validated knowledge scale, these findings should not be interpreted as demonstrating a formal knowledge–attitude–behavior gap.

Although the literature on premenstrual symptoms, self-medication, and complementary treatment practices is growing, these dimensions are often examined separately. Evidence from single-university settings comparing medication-related behaviors, healthcare-seeking practices, complementary product use, information sources, and medication-safety attitudes across academic faculties remains limited. In particular, it remains unclear whether students in health-related faculties consistently report different medication-related behaviors from those in non-health-related faculties, or whether favorable medication-safety attitudes coexist with self-medication-related practices.

Accordingly, this study aimed to describe medication-related, self-care, healthcare-seeking, and complementary-product behaviors among female university students reporting premenstrual symptoms and to examine whether these outcomes differed across five academic faculties. The specific research questions were as follows: (1) What medication-related and self-care practices are reported for premenstrual symptom management? (2) Do healthcare-seeking, medication-use, complementary-product, information-seeking, and medication-safety attitudes differ across the five faculties? and (3) To what extent do favorable medication-safety attitudes coexist with self-medication-related behaviors? Health-related versus non-health-related disciplines were considered an interpretive grouping, whereas the faculty-level comparison constituted the primary analytical framework.

## 2. Materials and Methods

### 2.1. Study Design and Setting

This cross-sectional study was conducted between 1 January and 30 June 2024 among female undergraduate students enrolled in the Faculties of Medicine, Dentistry, Education, Science and Literature, and Sports Sciences at Çukurova University, Türkiye. The study was designed and reported in accordance with the Strengthening the Reporting of Observational Studies in Epidemiology (STROBE) guidelines.

Ethical approval was obtained from the Çukurova University Faculty of Medicine Non-Interventional Clinical Research Ethics Committee (Decision No. 74; 8 December 2023). The study procedures were conducted in accordance with the ethical principles of the Declaration of Helsinki. Written informed consent was obtained from all participants before data collection.

### 2.2. Study Population and Sampling

The study population comprised 2489 female undergraduate students enrolled in the Faculties of Education, Science and Literature, Medicine, Dentistry, and Sports Sciences at Çukurova University during the 2023–2024 academic year.

The minimum required sample size was estimated using the Raosoft^®^ Sample Size Calculator (Raosoft, Inc., Seattle, WA, USA; web-based calculator, ©2004), assuming a 95% confidence level, a 5% margin of error, and a 50% response distribution; this yielded a reference minimum of 333 participants.

Recruitment relied on voluntary participation among eligible students during the study period. Trained researchers explained the study objectives and procedures face-to-face, after which students who agreed to participate provided written informed consent. A total of 500 students volunteered to participate (20.1% of the eligible population). After data screening, 21 questionnaires (4.2% of the volunteer sample) were excluded because of incomplete responses, leaving 479 participants (19.2% of the eligible population) for analysis. Thus, the final sample was a volunteer/convenience sample rather than a probability sample, and the Raosoft calculation was used only as a reference benchmark for the minimum sample size. Accordingly, the reported margin of error should not be interpreted as a probability-sampling precision estimate.

**Figure 1:**
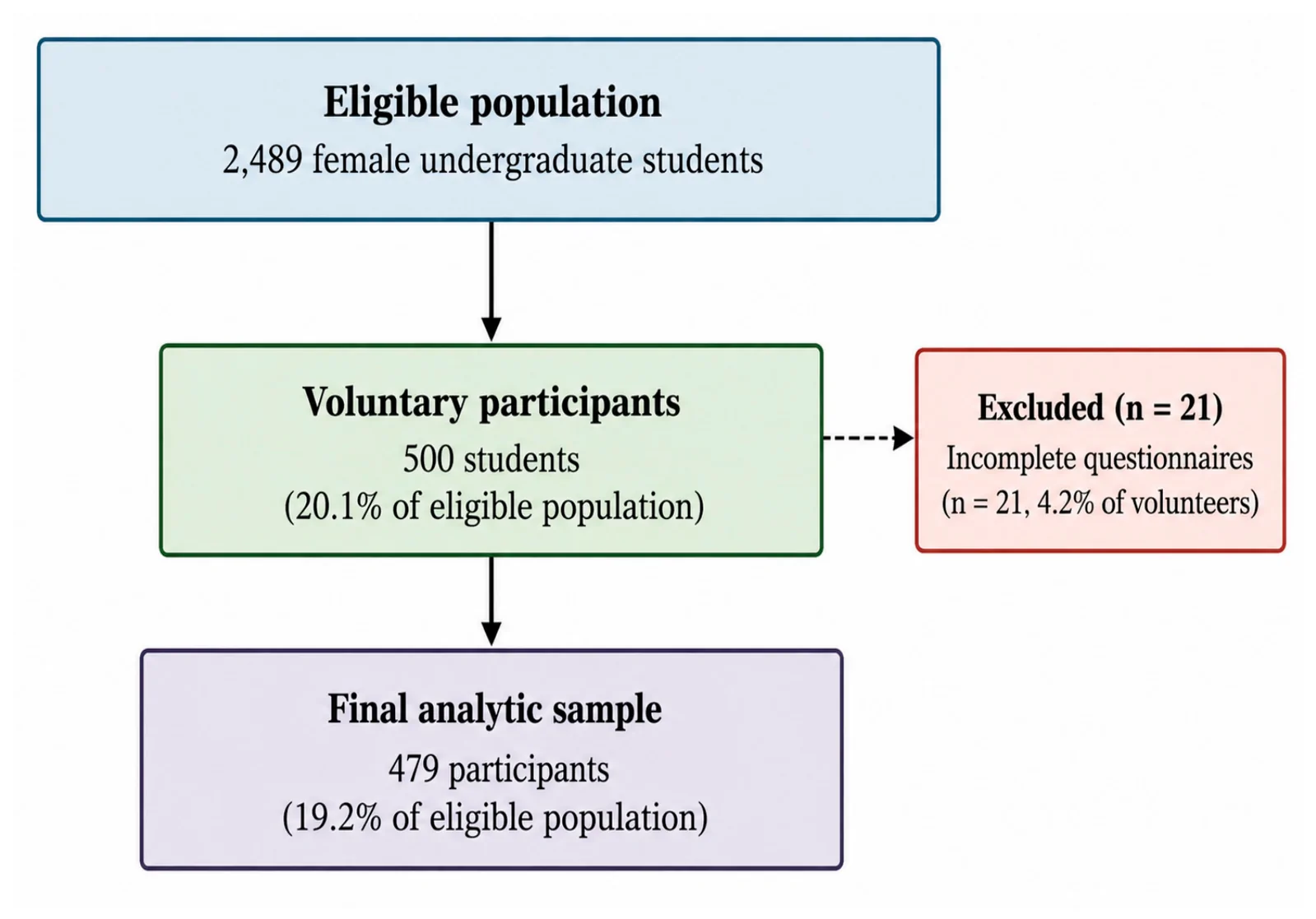
Flow diagram of participant recruitment and selection for the study. Of the 2489 eligible female undergraduate students, 500 volunteered to participate. After excluding 21 incomplete questionnaires, data from 479 participants were included in the final analyses.

Faculty-level analyses were prespecified as the primary analytical approach. Medicine and Dentistry were categorized as health-related disciplines and Education, Science and Literature, and Sports Sciences as non-health-related disciplines for descriptive interpretation only. These groupings were not treated as a causal measure of health-related education. Participant recruitment and exclusions are summarized in Figure 1.

### 2.3. Eligibility Criteria

The inclusion criteria were: (1) being a female undergraduate student aged ≥18 years; (2) self-reported experience of premenstrual symptoms; and (3) willingness to participate voluntarily and provision of informed consent. Participants who did not report premenstrual symptoms or who submitted incomplete questionnaires were excluded from the final analysis. No clinical assessment or diagnostic interview for premenstrual symptoms or PMDD was performed; therefore, the study population should be described as students reporting premenstrual symptoms rather than students with clinically diagnosed PMS or PMDD.

### 2.4. Data Collection Instrument

Data were collected using a structured questionnaire developed by the researchers based on a literature review addressing premenstrual symptom management, self-medication, medication safety, healthcare-seeking behavior, and complementary and herbal practices. The questionnaire consisted of 53 items organized into four sections. The first section included five items assessing the presence and characteristics of premenstrual symptoms. The second section included nine items addressing sociodemographic and lifestyle characteristics. The third section comprised 16 items assessing pharmacological and non-pharmacological approaches used for symptom management, including medication use, self-medication practices, healthcare-seeking behavior, and complementary approaches. The fourth section included 22 five-point Likert-scale items addressing attitudes and self-reported behaviors related to medication safety, professional healthcare guidance, informal sources of health information, and premenstrual symptom management. The questionnaire was administered face-to-face by trained researchers after the study objectives and procedures had been explained. The first three sections consisted primarily of closed-ended questions, whereas the fourth section used a five-point Likert response format. The average completion time was approximately 15 min. The instrument was developed as a study-specific descriptive questionnaire rather than a validated diagnostic or psychometric scale. No formal pilot testing, content-validity index, internal-consistency analysis, construct validation, or diagnostic cut-off was performed, and no overall medication-safety score was calculated.

### 2.5. Outcome Measures

The primary outcome domain was medication-related behavior during management of premenstrual symptoms, with particular emphasis on self-medication and analgesic use without physician recommendation. Secondary outcome domains included healthcare-seeking behavior, use of medications available at home, complementary and herbal product use, sources of health information, and attitudes related to medication safety and professional guidance. Differences in these outcomes were examined across the five academic faculties.

Medication-safety attitudes and behaviors were assessed through specific questionnaire items addressing physician consultation, self-medication, use of previously available medications, analgesic use without physician recommendation, reliance on informal treatment recommendations, and attitudes toward professional decision-making. Because the questionnaire was developed specifically for this study and was not a validated psychometric scale, no overall medication-safety or rational-drug-use score was calculated.

Statistical analyses were performed using IBM SPSS Statistics version 20.0 (IBM Corp., Armonk, NY, USA). Continuous variables were summarized using mean ± standard deviation (SD), while categorical variables were presented as frequencies (*n*) and percentages (%). Pearson’s chi-square test was used to examine associations between academic faculty and categorical outcomes, including healthcare-seeking behavior, self-medication practices, medication use, complementary and herbal product use, sources of health information, and medication-safety attitudes. Cramer’s V was reported to describe the magnitude of categorical associations. All statistical tests were two-sided, and *p* < 0.05 was considered statistically significant. No formal multiplicity adjustment was prespecified. Because numerous related outcomes were examined, *p*-values are presented as nominal, and the analyses are considered exploratory. Statistical significance was therefore interpreted together with effect size and the consistency of findings across related outcomes.

## 3. Results

### 3.1. Characteristics of the Study Population

A total of 479 female university students were included in the final analysis. Participants were enrolled in five academic disciplines: Education, Science and Literature, Medicine, Dentistry, and Sports Sciences. Most participants were aged 18–25 years (98.7%). The largest proportion of participants was enrolled in the Faculty of Education (23.2%), followed by the Faculties of Science and Literature (20.9%), Medicine (20.3%), Dentistry (20.0%), and Sports Sciences (15.7%).

Overall, 88.1% of participants reported regular menstrual cycles. Because self-reported premenstrual symptoms were an eligibility criterion, this percentage should not be interpreted as the prevalence of premenstrual symptoms in the wider student population. Previous education on premenstrual symptom management was reported by 30.5% of participants. The questionnaire did not collect sufficient detail to characterize the source or format of this education. The highest proportion reporting previous education was observed among medical students.

Regarding healthcare-seeking behavior, family members or friends were the most frequently reported source of advice (47.4%), followed by physicians (27.6%). Reported sources of healthcare advice varied across faculties.

The most frequently reported strategies for managing premenstrual symptoms were sleep (81.2%), heat application (71.2%), medication use (63.3%), and bathing or showering (57.4%). Less frequently reported strategies included listening to music (31.1%), walking (30.3%), and herbal approaches (29.9%). Detailed participant characteristics and symptom-management practices are presented in Table 1.

### 3.2. Healthcare-Seeking and Pharmacological Management

**Figure 2:**
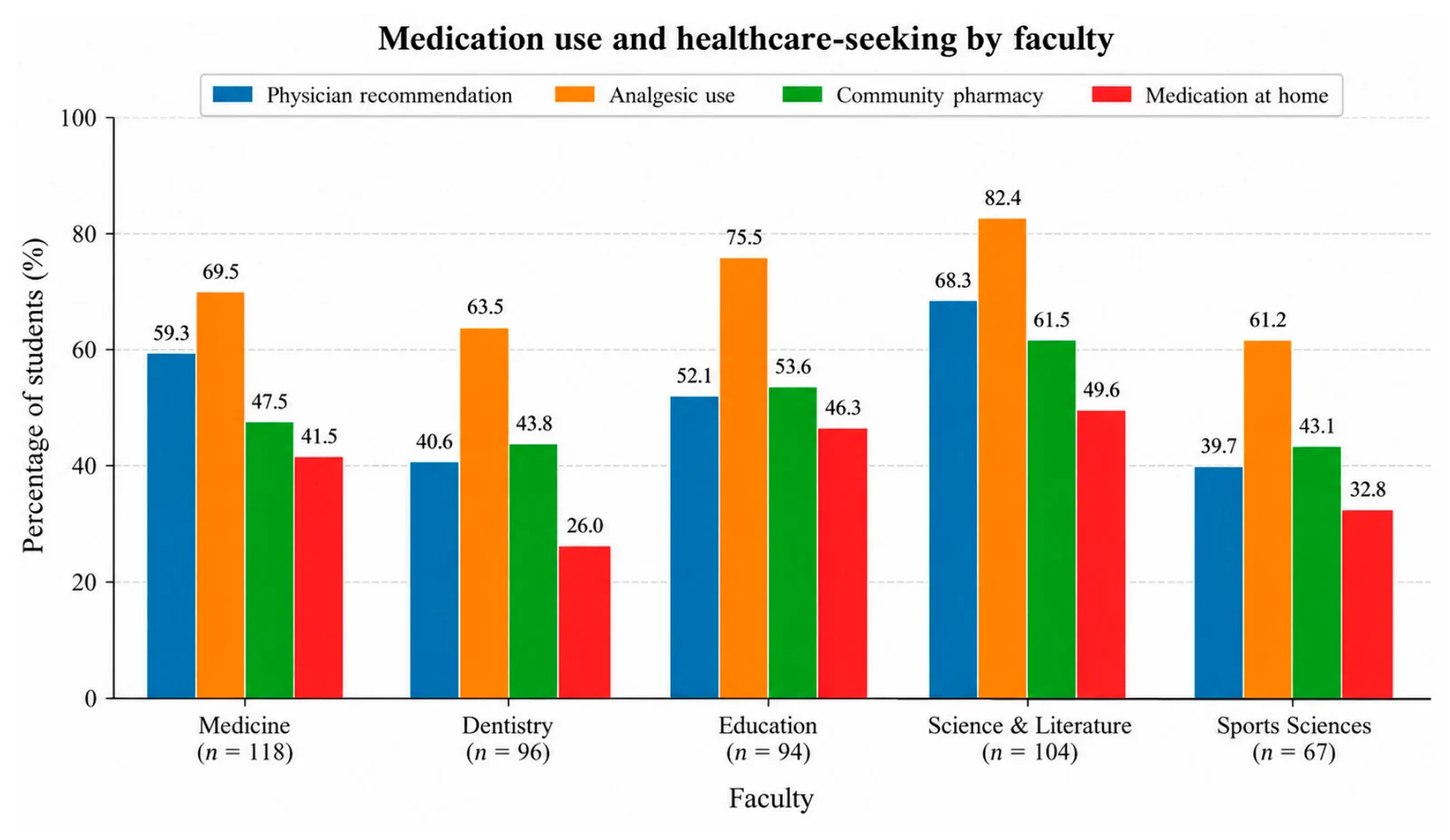
Faculty-specific patterns of medication use and healthcare-seeking for premenstrual symptom management. Values are presented as percentages within each faculty and were verified against Table 2.

Medication-related practices varied across the participating faculties (Table 2). Overall, 52.6% of participants reported using medications following a physician’s recommendation. The proportion varied across faculties, indicating differences in reported medication-use and healthcare-seeking practices. The faculty-specific distributions are visu-alized in Figure 2.

Analgesics were the most frequently reported medication category for managing premenstrual symptoms (71.0%). Use was highest among students in Science and Literature (82.0%) and Education (75.5%) and lower among students in Dentistry (63.5%) and Sports Sciences (61.3%).

Community pharmacies were the most frequently reported source of medications (50.5%). However, 39.9% of participants reported using medications already available at home. This practice was reported most often among students in Science and Literature (50.0%) and Education (45.9%).

### 3.3. Complementary and Non-Pharmacological Symptom Management

**Figure 3:**
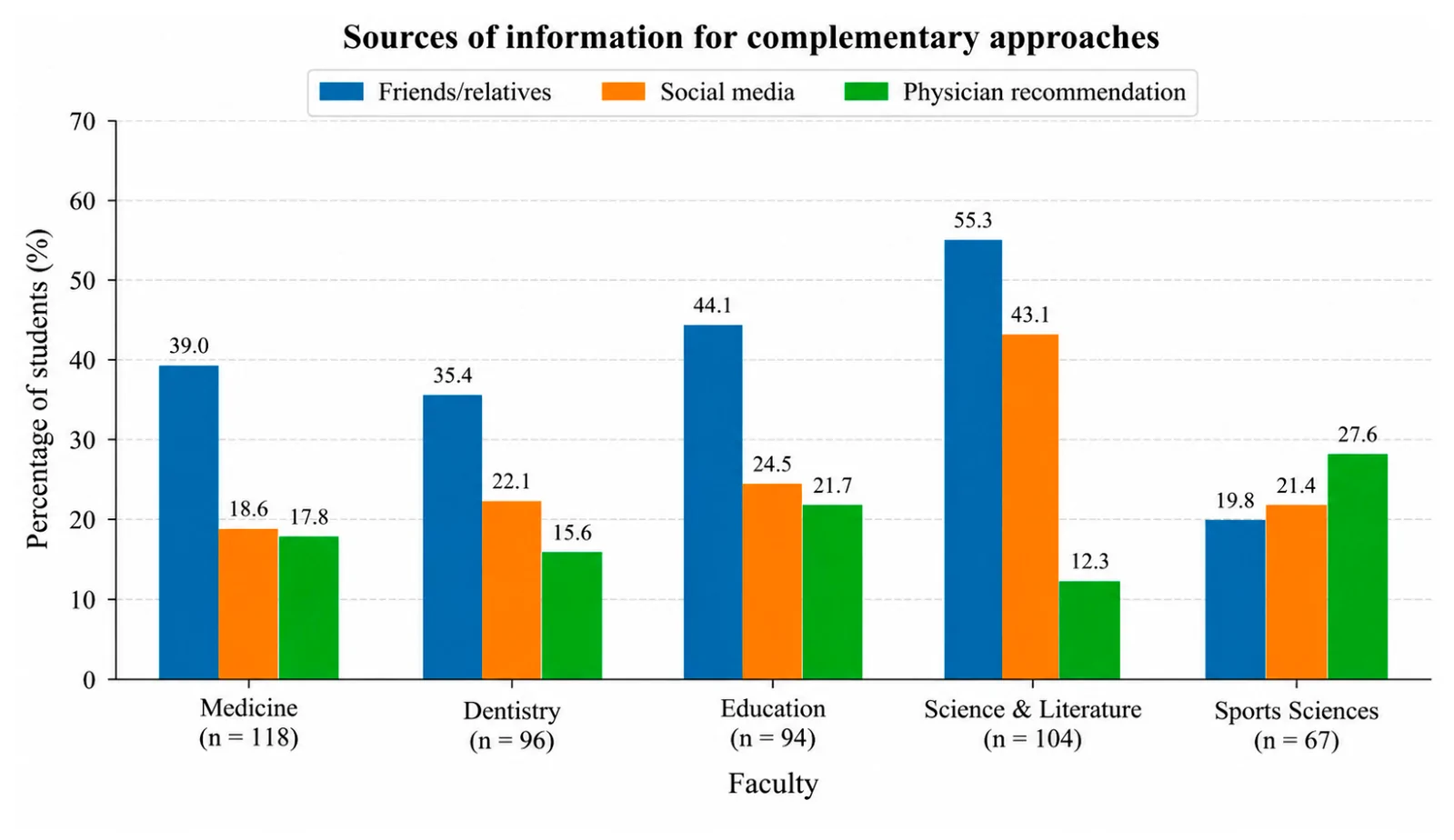
Sources of information for complementary and non-pharmacological approaches to premenstrual symptom management according to academic faculty. Values are presented as percentages within each faculty.

Non-pharmacological and complementary approaches were commonly reported among participants (Table 3). Friends or relatives were the most frequently reported source of information regarding complementary approaches (39.9%), followed by social media (26.1%) and physicians (18.6%). Information sources differed across faculties. Information sources differed across faculties. Faculty-specific information-source patterns are shown in Figure 3.

**Figure 4:**
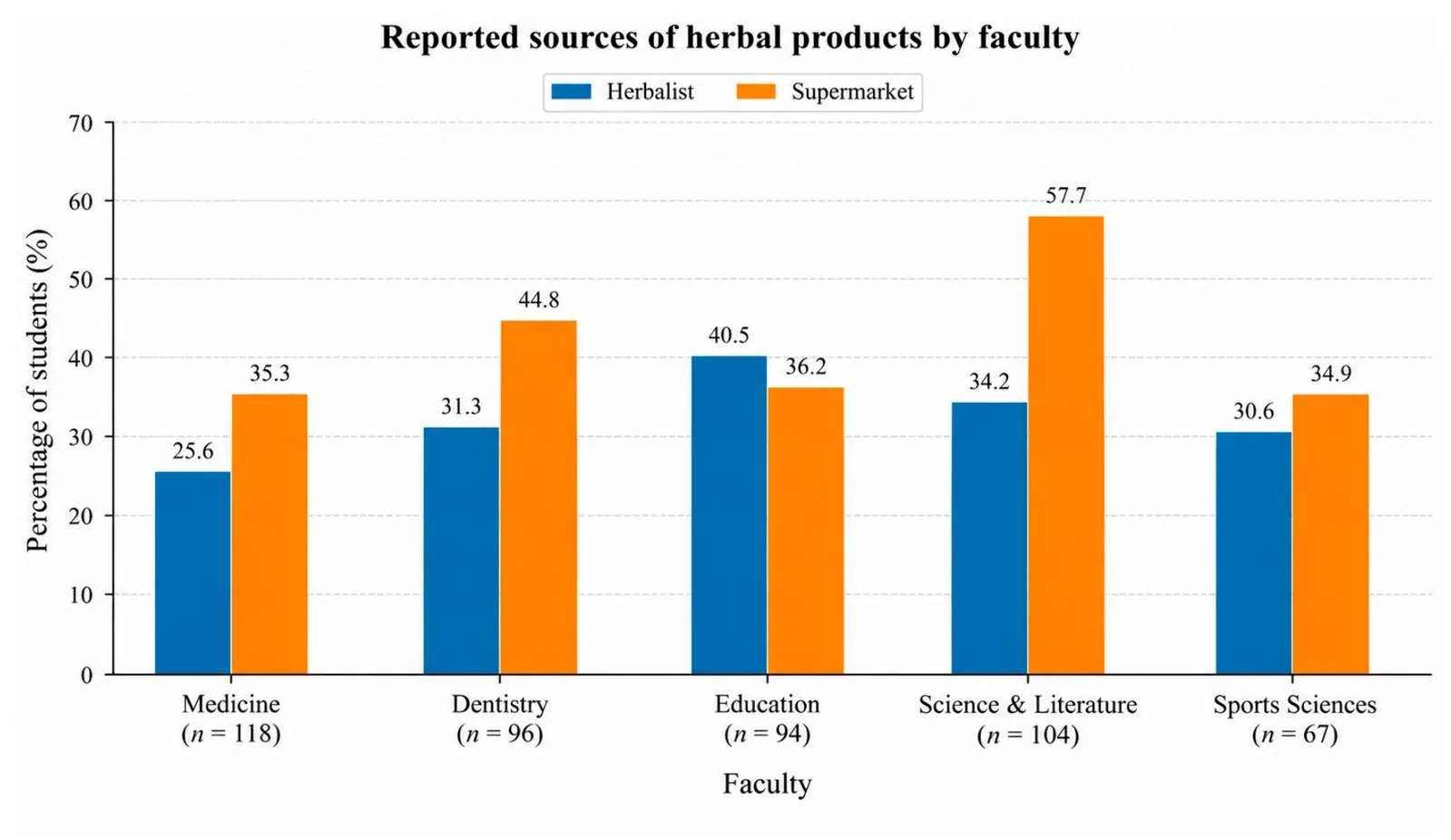
Sources of herbal products according to faculty. Values represent the reported proportions obtaining herbal products from herbalists and supermarkets.

Overall, 29.9% of participants reported using herbal or aromatherapy-based approaches for premenstrual symptoms. Use was highest among students in Science and Literature (45.0%) and lowest among Sports Sciences students (13.3%). Among participants reporting herbal product use, supermarkets (42.0%) and herbalists (32.8%) were the most frequently reported sources. Faculty-specific source patterns are shown in Figure 4.

The most frequently reported herbal products were green tea (35.3%), chamomile (31.7%), and linden (26.9%), followed by fennel (15.2%) and ginger (14.0%). Lavender oil was the most frequently reported aromatherapy product (6.5%).

Overall, participants used a combination of pharmacological, non-pharmacological, and complementary approaches to manage premenstrual symptoms. However, the use of complementary approaches was not necessarily accompanied by professional guidance.

### 3.4. Attitudes Toward Medication Safety and Complementary Approaches

**Figure 5:**
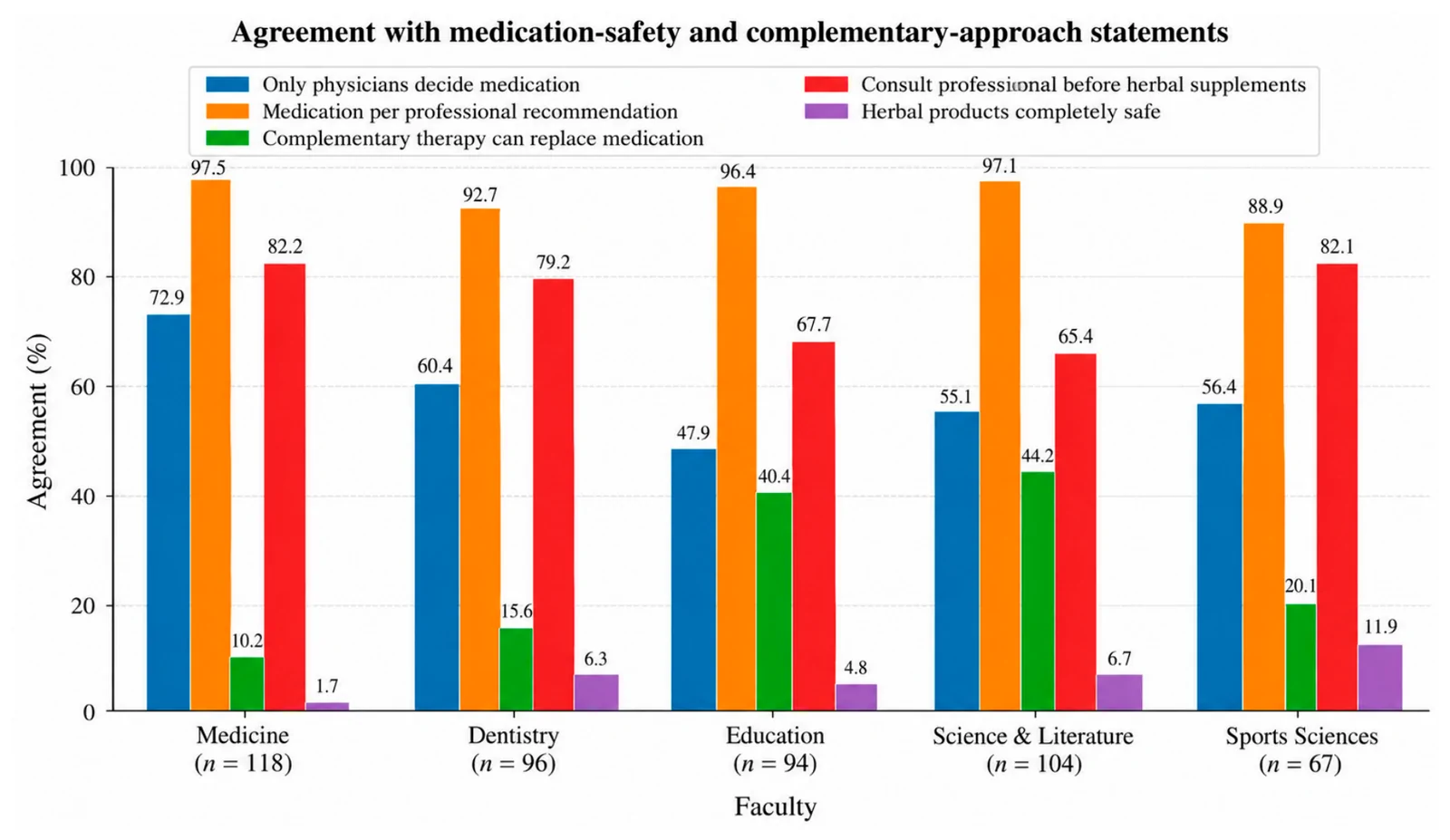
Faculty-specific attitudes toward medication and herbal supplements in premenstrual symptom management. Six statements are shown across the five faculties; responses are presented as percentages of agree, neutral, and disagree categories.

Participants generally expressed favorable attitudes toward professional guidance and medication safety; however, several attitudes differed across academic faculties (Table 4). The distribution of agreement responses is summarized in Figure 5.

The highest proportion agreeing that only physicians should decide whether medication is required for premenstrual symptoms was observed among medical students (73.2%). Medical students also showed a comparatively cautious attitude toward complementary approaches and were less likely to regard complementary therapies as substitutes for conventional medication. They also showed the strongest rejection of the statement that herbal products are completely safe and can be consumed without restriction.

Across faculties, attitudes toward replacing conventional medication with traditional or complementary approaches varied substantially. Students in Science and Literature and Education showed higher agreement with the possibility that complementary therapies could replace conventional medication, whereas medical students showed greater disagreement.

Attitudes toward consulting healthcare professionals before using herbal supplements also differed across faculties. These findings describe differences in perceptions of medication safety and professional guidance across academic faculties; they do not establish that academic education caused these differences.

### 3.5. Self-Medication and Information-Seeking Behaviors

Self-medication-related behaviors differed across academic faculties (Table 5). For the statement regarding use of medications already available at home to accelerate symptom relief, agreement was 68.0% in Science and Literature, 70.9% in Education, 45.8% in Dentistry, 58.8% in Medicine, and 64.0% in Sports Sciences. These values are consistent with Table 5; the original text had the faculty values misassigned.

Analgesic use without physician recommendation was frequently reported across faculties, with agreement ranging from 57.3% in Dentistry to 78.4% in Medicine.

The use of herbal products without physician recommendation also varied across faculties, with the highest agreement in Science and Literature (70.0%) and the lowest in Sports Sciences (34.7%) and Medicine (35.1%).

Informal recommendations also contributed to symptom-management decisions. Agreement that methods recommended by people around the participant were more effective ranged from 3.1% in Medicine to 32.0% in Sports Sciences, indicating substantial faculty-level variation.

Social media represented an important source of health-related information, although agreement that solutions learned from social media were more effective was low in all faculties, ranging from 3.1% in Medicine to 10.9% in Education.

Overall, self-medication-related and informal information-seeking behaviors were reported across academic faculties, including among students in health-related disciplines.

## 4. Discussion

This study examined medication-related behavior, healthcare-seeking practices, self-medication, complementary and herbal product use, and medication-safety attitudes among female university students reporting premenstrual symptoms. Faculty-level differences were observed across several outcomes, while self-medication-related practices remained common in both health-related and non-health-related faculties.

A central finding was the coexistence of favorable medication-safety attitudes with persistent self-medication-related behaviors. Medical students reported more cautious attitudes toward several herbal and informal-information practices, yet they also reported analgesic use without physician recommendation and use of previously available medications. Because the questionnaire was not a validated knowledge scale, these findings should not be interpreted as demonstrating a formal knowledge–attitude–behavior gap.

Previous education on premenstrual symptom management was reported by 30.5% of participants. The study did not capture sufficient detail about the source or format of this education to determine whether it represented a course, counseling session, workshop, or another educational activity. Accordingly, the observed faculty differences should be interpreted descriptively rather than as evidence that formal education caused safer behavior.

Analgesics were the most frequently reported medication category (71.0%), and analgesic use without physician recommendation was common across faculties, including among medical students. Use without physician recommendation should not be equated automatically with irrational or inappropriate use because the study did not assess indication, dose, duration, contraindications, or interactions for individual medication episodes. This finding should therefore be interpreted as a medication-use behavior with potential safety implications rather than as a measure of inappropriate use.

Use of medications already available at home was reported by 39.9% of participants. This behavior may reflect convenience or previous experience, but the appropriateness of a previously obtained medicine depends on the current indication, dosage, expiration date, contraindications, comorbidities, and potential interactions with other medicines [22,23]. Home-stored medication use is therefore an important self-medication-related behavior that warrants attention in university health promotion.

Non-pharmacological approaches were also common. Sleep, heat application, medication use, and bathing were among the most frequently reported strategies, while walking, listening to music, and herbal approaches were also used. These findings suggest that participants used multiple management strategies rather than relying exclusively on medication. Taking rest and sleeping have been reported as supportive coping mechanisms for premenstrual symptoms [28]. Evidence also supports the potential role of physical activity in symptom management [29]. Music has likewise been described as a beneficial non-pharmacological approach [30].

Nearly one-third of participants reported herbal or aromatherapy-based approaches, with green tea, chamomile, linden, fennel, and ginger among the most frequently reported products [31,32,33,34,35]. Herbal products were commonly obtained from supermarkets or herbalists. Their natural origin should not be interpreted as evidence of safety; product composition, dosage, quality, contamination, and interactions with conventional medicines may affect risk [24,25].

An important finding was the contrast between attitudes toward herbal safety and actual herbal-product use. Medical students were more likely to reject the statement that herbal products are completely safe and can be consumed without restriction, yet herbal-product use without physician recommendation remained common. This pattern is consistent with the broader observation that favorable attitudes do not necessarily predict reported behavior.

Friends or relatives were the most frequently reported source of information regarding complementary approaches, followed by social media and physicians. This pattern indicates that symptom-management decisions occur within a social and digital information environment. Previous studies have similarly shown that family, friends, and online sources can influence complementary medicine use and health-related help-seeking [36,37,38,39].

Faculty-level findings did not show a uniform behavioral pattern. Medical students tended to report more cautious attitudes toward herbal safety and social-media-derived recommendations, whereas some non-health-related faculties showed greater reliance on informal recommendations or herbal products. These associations may also reflect differences in symptom burden, age, previous experience, medication accessibility, social influence, and healthcare access; none of these factors was controlled for in the present analyses.

The findings support university-based health promotion that moves beyond information provision toward practical medication-safety skills. Programs should strengthen recognition of when professional assessment is needed, safe use of medicines, avoidance of inappropriate reuse of previously obtained medicines, evaluation of digital health information, and informed decisions about complementary and herbal products.

### Limitations

This study has several limitations. First, recruitment relied on voluntary participation at a single university, which may have introduced selection and voluntary-response bias and limits generalizability beyond the sampled faculties and institutional setting. Second, premenstrual symptoms were self-reported and were not confirmed using established diagnostic criteria or clinical assessment; therefore, the findings should not be generalized to clinically diagnosed premenstrual symptoms or PMDD. Third, the questionnaire was developed for this study and was not psychometrically validated, and no formal pilot testing or reliability assessment was performed. Fourth, the study did not collect sufficient clinical detail on individual medication episodes (e.g., indication, dose, duration, contraindications, and interactions), so medication use without physician recommendation cannot be classified as irrational or inappropriate. Fifth, the analyses were unadjusted and did not account for potential confounders such as age, year of study, socioeconomic status, healthcare access, symptom severity, or previous education. Finally, no formal adjustment for multiple comparisons was prespecified; reported *p*-values should therefore be interpreted as nominal and hypothesis-generating.

## 5. Conclusions

This study identified substantial use of self-management and medication-related practices among female university students reporting premenstrual symptoms, with variation across academic faculties. Students in health-related faculties, particularly medical students, reported more cautious attitudes toward several complementary and herbal practices, but self-medication-related behaviors remained common.

The findings do not establish that academic education caused these differences, and the study cannot determine the appropriateness of individual medication use. University-based health promotion should therefore focus on practical medication-safety skills, appropriate healthcare seeking, evidence-based self-care, responsible use of complementary products, and critical evaluation of informal and digital health information.

An integrated approach involving university health services, healthcare professionals, and health promotion programs may support safer and more informed management of premenstrual symptoms within the university setting.

## Figures and Tables

**Table 1:** Sociodemographic characteristics, lifestyle factors, and premenstrual symptom-management practices among female university students (*n* = 479).

Characteristic	*n*	%
Age		
18–25 years	473	98.7
Faculty		
Education	111	23.2
Science and Literature	100	20.9
Medicine	97	20.3
Dentistry	96	20.0
Sports Sciences	75	15.7
Lifestyle characteristics		
Current smoker	99	20.7
Alcohol consumption	163	34.0
Regular menstrual cycles	422	88.1
Previous education on premenstrual symptom management	146	30.5
First source of consultation for premenstrual symptom management *		
Family/Friends	227	47.4
Physician	132	27.6
Most frequently used premenstrual symptom management strategies *		
Sleep	389	81.2
Heat application	341	71.2
Medication use	303	63.3
Bath/Shower	275	57.4
Listening to music	149	31.1
Walking	145	30.3
Herbal therapies	143	29.9

* Multiple responses were permitted; percentages therefore do not sum to 100%.

**Table 2:** Medication use and healthcare-seeking practices for premenstrual symptoms according to academic faculty (*n* = 479).

Variable	Total *n* (%)	Medicine *n* (%)	Dentistry *n* (%)	Education *n* (%)	Science and Literature *n* (%)	Sports Sciences *n* (%)	*p*-Value	Cramer’s V
Medication use following physician recommendation	252 (52.6)	57 (58.8)	39 (40.6)	58 (52.3)	68 (68.0)	30 (40.0)	0.005	0.211
Analgesic use	340 (71.0)	67 (69.1)	61 (63.5)	84 (75.5)	82 (82.0)	46 (61.3)	0.008	0.166
Medication obtained from community pharmacies	242 (50.5)	46 (47.4)	42 (43.8)	60 (54.1)	62 (62.0)	32 (42.7)	0.008	0.143
Medications already available at home	191 (39.9)	40 (41.2)	25 (26.0)	51 (45.9)	50 (50.0)	25 (33.3)	0.005	0.177

Values are presented as *n* (%). Differences between faculties were evaluated using Pearson’s chi-square test. Cramer’s V was used to quantify the magnitude of categorical associations.

**Table 3:** Faculty-level comparisons of complementary and non-pharmacological approaches to premenstrual symptom management among female university students (*n* = 479).

Variable	Total *n* (%)	Medicine *n* (%)	Dentistry *n* (%)	Education *n* (%)	Science and Literature *n* (%)	Sports Sciences *n* (%)	χ^2^	*p*-Value	Cramer’s V
Information source for complementary methods									
Friends/relatives	191 (39.9)	38 (39.2)	34 (35.4)	49 (44.1)	55 (55.0)	15 (20.0)	32.908	*p* < 0.001	0.262
Social media	125 (26.1)	18 (18.6)	21 (21.9)	27 (24.3)	43 (43.0)	16 (21.3)	24.172	*p* < 0.001	0.225
Physician recommendation	89 (18.6)	17 (17.5)	15 (15.6)	24 (21.6)	12 (12.0)	21 (28.0)	11.535	*p* = 0.021	0.155
Complementary therapy use									
Herbal/Aromatherapy use	143 (29.9)	26 (26.8)	25 (26.0)	37 (33.3)	45 (45.0)	10 (13.3)	29.462	*p* < 0.001	0.248
Source of herbal products									
Herbalist	157 (32.8)	25 (25.8)	30 (31.3)	45 (40.5)	34 (34.0)	23 (30.7)	13.450	*p* = 0.009	0.168
Supermarket	201 (42.0)	34 (35.1)	43 (44.8)	40 (36.0)	58 (58.0)	26 (34.7)	17.384	*p* = 0.002	0.191

Values are presented as *n* (%). Multiple responses were permitted for information sources, management strategies, and herbal-product sources where applicable. Pearson’s chi-square test and Cramer’s V were used for faculty-level comparisons.

**Table 4:** Attitudes toward medication safety, professional guidance, and complementary approaches to premenstrual symptom management according to academic faculty (*n* = 479).

Statement	Response	Science & Literature (%)	Education (%)	Dentistry (%)	Medicine (%)	Sports Sciences (%)	χ^2^	*p*	Cramer’s V
Only physicians should decide whether medication is required for premenstrual symptom treatment.	Agree	55.0	47.3	60.4	73.2	56.0	18.852	0.016	0.140
	Neutral	33.0	34.5	24.0	15.5	24.0			
	Disagree	12.0	18.2	15.6	11.3	20.0			
Medications should always be used according to physician/pharmacist recommendations.	Agree	97.0	96.4	92.7	96.9	89.3	11.833	0.159	0.111
	Neutral	3.0	2.7	3.1	2.1	8.0			
	Disagree	0.0	0.9	4.2	1.0	2.7			
Traditional and complementary medicine can replace conventional medication for premenstrual symptoms.	Agree	44.0	40.0	15.6	10.3	20.0	61.534	<0.001	
	Neutral	30.0	23.6	43.8	26.8	42.7			
	Disagree	26.0	36.4	40.6	62.9	37.3			
A healthcare professional should always be consulted before using herbal supplements.	Agree	65.0	68.2	79.2	82.5	82.7	17.208	0.028	0.253
	Neutral	26.0	20.9	16.7	14.4	14.7			
	Disagree	9.0	10.9	4.2	3.1	2.7			
Herbal supplements purchased from unknown sources may contain harmful substances.	Agree	90.0	82.7	80.2	86.6	85.3	8.954	0.346	0.134
	Neutral	8.0	12.7	9.4	8.2	9.3			
	Disagree	2.0	4.5	10.4	5.2	5.3			
Herbal products are completely safe and can be consumed without restriction.	Agree	6.0	4.5	6.3	0.0	12.0	53.579	<0.001	
	Neutral	4.0	12.7	8.3	2.1	28.0			
	Disagree	90.0	82.7	85.4	97.9	60.0			

Values are presented as percentages within each faculty. Differences were evaluated using Pearson’s chi-square test. Cramer’s V was used to assess the magnitude of categorical associations.

**Table 5:** Self-medication and informal information-seeking behaviors for premenstrual symptom management according to academic faculty (*n* = 479).

Statement	Response	Science & Literature (%)	Education (%)	Dentistry (%)	Medicine (%)	Sports Sciences (%)	χ^2^	*p*	Cramer’s V
I use medications already available at home to accelerate symptom relief.	Agree	68.0	70.9	45.8	58.8	64.0	20.459	0.009	0.146
	Neutral	11.0	7.3	22.9	13.4	9.3			
	Disagree	21.0	21.8	31.3	27.8	26.7			
I use analgesics without physician recommendation.	Agree	70.0	74.5	57.3	78.4	60.0	25.960	0.001	0.165
	Neutral	3.0	8.2	16.7	6.2	18.7			
	Disagree	27.0	17.3	26.0	15.5	21.3			
I use herbal products without physician recommendation.	Agree	70.0	52.7	47.9	35.1	34.7	43.379	<0.001	
	Neutral	9.0	14.5	17.7	13.4	30.7			
	Disagree	21.0	32.7	34.4	51.5	34.7			
Methods recommended by people around me are more effective.	Agree	32.0	31.8	9.4	3.1	17.3	57.571	<0.001	
	Neutral	23.0	25.5	30.2	18.6	36.0			
	Disagree	45.0	42.7	60.4	78.4	46.7			
Solutions learned from social media are more effective.	Agree	6.0	10.9	4.2	3.1	9.3	26.992	0.001	0.213
	Neutral	13.0	16.4	15.6	6.2	29.3			
	Disagree	81.0	72.7	80.2	90.7	61.3			

Values are presented as percentages within each faculty. Differences were evaluated using Pearson’s chi-square test. Cramer’s V was used to assess the magnitude of categorical associations.

## Data Availability

The data supporting the findings of this study are available from the corresponding author upon reasonable request. The data are not publicly available due to privacy and ethical restrictions.
